# Diaphragm Abnormalities in Patients with End-Stage Heart Failure: NADPH Oxidase Upregulation and Protein Oxidation

**DOI:** 10.3389/fphys.2016.00686

**Published:** 2017-01-09

**Authors:** Bumsoo Ahn, Philip D. Coblentz, Adam W. Beharry, Nikhil Patel, Andrew R. Judge, Jennifer. S. Moylan, Charles W. Hoopes, Mark R. Bonnell, Leonardo F. Ferreira

**Affiliations:** ^1^Department of Applied Physiology and Kinesiology, University of FloridaGainesville, FL, USA; ^2^Department of Physical Therapy, University of FloridaGainesville, FL, USA; ^3^Department of Physiology, University of KentuckyLexington, KY, USA; ^4^Division of Cardiothoracic Surgery, University of Alabama at BirminghamBirmingham, AL, USA; ^5^Division of Cardiothoracic Surgery, University of Toledo Medical CenterToledo, OH, USA

**Keywords:** diaphragm, carbonyls, weakness, inspiratory muscles, NOX2

## Abstract

Patients with heart failure (HF) have diaphragm abnormalities that contribute to disease morbidity and mortality. Studies in animals suggest that reactive oxygen species (ROS) cause diaphragm abnormalities in HF. However, the effects of HF on ROS sources, antioxidant enzymes, and protein oxidation in the diaphragm of humans is unknown. NAD(P)H oxidase, especially the Nox2 isoform, is an important source of ROS in the diaphragm. Our main hypothesis was that diaphragm from patients with HF have heightened Nox2 expression and p47^phox^ phosphorylation (marker of enzyme activation) that is associated with elevated protein oxidation. We collected diaphragm biopsies from patients with HF and brain-dead organ donors (controls). Diaphragm mRNA levels of Nox2 subunits were increased 2.5–4.6-fold over controls (*p* < 0.05). Patients also had increased protein levels of Nox2 subunits (p47^phox^, p22^phox^, and p67^phox^) and total p47^phox^ phosphorylation, while phospho-to-total p47^phox^ levels were unchanged. The antioxidant enzyme catalase was increased in patients, whereas glutathione peroxidase and superoxide dismutases were unchanged. Among markers of protein oxidation, carbonyls were increased by ~40% (*p* < 0.05) and 4-hydroxynonenal and 3-nitrotyrosines were unchanged in patients with HF. Overall, our findings suggest that Nox2 is an important source of ROS in the diaphragm of patients with HF and increases in levels of antioxidant enzymes are not sufficient to maintain normal redox homeostasis. The net outcome is elevated diaphragm protein oxidation that has been shown to cause weakness in animals.

## Introduction

Patients with end-stage heart failure (HF) have respiratory complications that contribute to disease morbidity and mortality. The incidence of pneumonia in end-stage HF patients who receive a heart transplant is 15–20% and 5% of patients have prolonged respiratory failure post-heart transplant (Lenner et al., 2001). The cause of pneumonia and respiratory failure in end-stage HF patients is multifactorial. In this context, inspiratory muscle abnormalities in patients with end-stage HF can play a critical role in respiratory complications (Kelley and Ferreira, 2016). For instance, pre-operative inspiratory muscle strength training reduces the incidence of post-operative pulmonary complications in “high-risk” patients, including those with HF, undergoing coronary artery bypass graft (Hulzebos et al., 2006).

The primary inspiratory muscle is the diaphragm, which is necessary for normal ventilatory and expulsive behaviors that promote airway clearance (Sieck and Fournier, 1989; Mantilla and Sieck, 2013). Diaphragm dysfunction has been documented in animal models of HF, being characterized by atrophy and contractile impairments that diminish force and power capabilities (Howell et al., 1995; Stassijns et al., 1998; van Hees et al., 2007; Ahn et al., 2015; Kelley and Ferreira, 2016). The diaphragm of end-stage HF patients also shows ultra-structural and myofibrillar protein alterations that suggest metabolic and contractile abnormalities (Lindsay et al., 1996). However, less is known about the underlying mechanisms of diaphragm dysfunction in end-stage HF.

Excess reactive oxygen species (ROS) and redox imbalance play a causal role in diaphragm dysfunction in animal models of HF (Supinski and Callahan, 2005; Ahn et al., 2015). An important source of ROS in the diaphragm is the Nox2 isoform of NAD(P)H oxidase (Pal et al., 2013; Loehr et al., 2014; Bost et al., 2015). A functionally assembled Nox2 enzyme complex consists of several subunits (Nox2, p22^phox^, p67^phox^, p40^phox^, Rac1/2, and p47^phox^), and enzyme activation requires p47^phox^ phosphorylation (Javesghani et al., 2002; Lassègue et al., 2012; Pal et al., 2013; Ferreira and Laitano, 2016). Diaphragm p47^phox^ phosphorylation is increased in mice with HF, and genetic deletion of p47^phox^ prevents excess ROS release and contractile dysfunction in diaphragm of mice with HF (Ahn et al., 2015). These findings in mice suggest a pivotal role for Nox2-derived ROS on diaphragm dysfunction in HF.

Diaphragm antioxidant enzymes are unchanged (Ahn et al., 2015) or elevated in animal models of HF (Bowen et al., 2015b; Mangner et al., 2015). The increase in antioxidant enzymes may reflect a compensatory response to scavenge excess ROS. However, these compensatory responses appear insufficient to maintain cellular redox homeostasis as markers of protein oxidation are elevated in diaphragm of HF animals (acute Bowen et al., 2015a and chronic models Supinski and Callahan, 2005; Coirault et al., 2007). This is relevant because heightened ROS and protein oxidation promote diaphragm atrophy and impair contractile function (Ferreira and Reid, 2008; Powers et al., 2011).

Despite advances in understanding the causes and role of redox imbalance on diaphragm dysfunction in animal models of HF, very little is known about ROS sources, antioxidant enzymes, and protein oxidation in the diaphragm of patients. We tested the hypotheses that diaphragm from patients with end-stage HF show heightened Nox2 subunit levels and p47^phox^ phosphorylation with unchanged protein levels of antioxidant enzymes which results in elevated markers of protein oxidation.

## Methods

### Human subjects

We obtained diaphragm biopsies from patients with HF with reduced ejection fraction who underwent surgery for heart transplant or placement of left ventricular assist device. Patients were informed of the nature and purpose of the study and signed a written consent in accordance with the Declaration of Helsinki. Control subjects were brain-dead organ donors whose family consented to the diaphragm biopsies. The protocol and sample analyses were approved by the Institutional Review Boards of the University of Kentucky or University of Florida.

### Tissue collection

The cardiothoracic surgeons (MRB and CWH) obtained diaphragm biopsies and placed them in ice- cold sterile saline. The samples were rapidly processed in the operating room to clear any visible connective tissue and excess blood, then frozen in liquid nitrogen and stored at −80°C for further processing as described below. All procedures for tissue collection and analyses were approved by the Institutional Review Boards of the University of Kentucky or University of Florida.

### qPCR

We isolated total RNA from human diaphragm tissue with Trizol reagent. We then used Ambion RETROscript First Strand Synthesis Kit (Life Technologies, Carlsbad, CA, USA) to generate cDNA from 1 μg of RNA. The cDNA was then used as template for qRT-PCR (7300 real-time PCR system, Applied Biosystems, Austin, TX). We used TaqMan® PCR assay primers from Life Technologies targeting the following genes and NCBI Reference Sequence numbers: Nox2 (*CYBB*, NM_000397.3), p47^phox^ (*NCF1* NM_000265.5), Rac1 (*RAC1*, NM_006908.4), p22^phox^ (*CYBA*, NM_000101.3), p67^phox^ (*NCF2*, NM_000433.3), and p40^phox^ (*NCF4*, NM_000631.4). Gene expression quantification was performed using the relative standard-curve method, and all data were normalized to the gene expression of *18S* (GeneBank NM_X03205.1) and reported relative to the control group.

### Immunoblotting

We loaded ~10–50 μg of protein into 4–20% stain-free TGX gels (Bio-Rad Laboratories) and performed electrophoresis at 230 V for 40 min on ice. We scanned the gel to quantify total proteins (Gel DocTM EZ Imager, Bio-Rad Laboratories) and then transferred the proteins to a nitrocellulose membrane at 100 mA overnight at 4°C. We blocked the membrane using Li-COR Blocking Buffer for 1 h at room temp and subsequently probed with primary antibodies. As markers of protein oxidation, we measured protein carbonyls (OxySelectTM Protein Carbonyl Immunoblot kit, Cell Biolabs), 4-hydroxynonenal (4-HNE, Ab46545, AbCam) adducts, and 3-nitrotyrosines (3-NT, 189542, Cayman Chemical). To probe for sources of ROS, we used primary antibodies targeting Nox2 (CYBB, 1:500 dilution, sc-5827, Santa Cruz), p22^phox^ (CYBA, 1:50 dilution, FL-195, Santa Cruz), p67^phox^ (NCF2, 1:50 dilution, sc-7663, Santa Cruz), Rac1 (RAC1, 1:1000 dilution 05-389, Millipore), p47^phox^ (NCF1, 1:1000 diltuion, SAB2500674, Sigma-Aldrich), and phosphorylated p47^phox^ at serine residues 345 (orb126026, Biobyrt), 370 (A1171, Assay Biotech), 359 (A1172, Assay Biotech), 328 (A1161, Assay Biotech), and 304 (A1160, Assay Biotech). The dilution for antibodies targeting serine residues was 1:1000. For antioxidant enzymes, we used antibodies targeting superoxide dismutase isoform 1 (SOD1; 1:500 dilution, FL-154, Santa Cruz), SOD2 (1:500 dilution, FL-122, Santa Cruz), catalase (1:1000 dilution, Ab16731, Abcam), and glutathione peroxidase (1:1000 dilution, Ab108427, Abcam). We diluted the primary antibodies in LiCor Blocking Buffer, incubated the membranes for 72 h at 4°C or 1 h at room temperature, and washed in TBS-T (Tris-buffered saline with 0.1% Tween) 4 × 5 min each. We then incubated the membranes in secondary antibodies (IR Dye, LI-COR) in Li-COR Blocking Buffer for 1 h at room temp, followed by 3 × 5 min washes in TBS-T and a 5 min rinse in TBS. We dried the membranes in an incubation chamber at ~37°C for 15 min and scanned the fluorescence signal using an Odyssey Infrared Imaging system (LI-COR, Lincoln, NE). We quantified the immunoblot signal using Image Studio Lite® (Li-COR) and the abundance of total protein in each gel lane using ImageLab (Bio-Rad Laboratories). The immunoblot signal of each target protein was normalized to the total protein measured in corresponding gel lanes. These procedures are consistent with recent recommendations for data analysis of Western blots using fluorescence methods and stain- free gels (Eaton et al., 2013; Murphy and Lamb, 2013).

### Statistical analysis

We performed statistical analysis using SigmaPlot v.12.5 (Systat Software, San Jose, CA). For specific comparisons, we used *t*-test or Mann-Whitney rank sum test for data that failed normality (Shapiro-Wilk test). Non-parametric data are presented as median ± interquartile range and shown in box and whisker plots. We declared statistical significance when *P* < 0.05.

## Results

Patient characteristics are detailed in Table 1. In summary, patients exhibited HF caused by ischemic (*n* = 5) and non-ischemic cardiomyopathy (*n* = 6).

**Table 1:** **Patient characteristics**.

	**Control**	**HF**
Age (year)	29 ± 6	50 ± 5^*^
Body weight (kg)	87 ± 15	90 ± 7
Height (m)	1.65 ± 0.01	1.79 ± 0.03
Ejection fraction (%)	–	20 (9)^*^
Males/Females	0/3	10/1

*HF, heart failure. Data are mean ± SE. Ejection fraction is shown as median (interquartile range) from 3 controls and 6 patients*.

* *P < 0.05 compared to control*.

**Figure 1:**
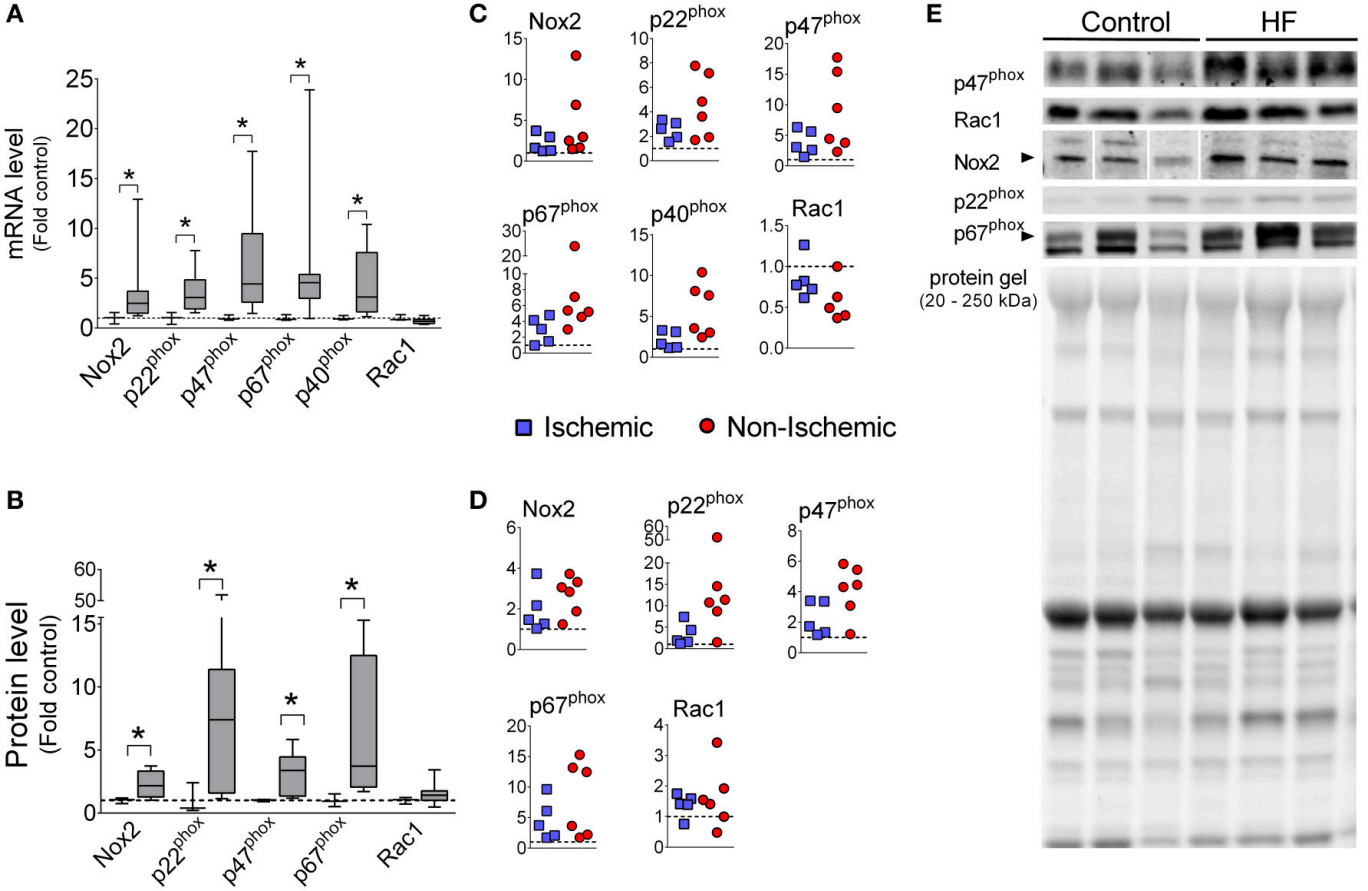
**Diaphragm Nox2 mRNA and protein levels are increased in patients with end-stage HF**. mRNA levels **(A,C)** and protein **(B,D)** of Nox2 subunits. Controls (open boxes; *n* = 3) and HF patients (gray boxes; *n* = 9–11). **(C,D)** are individual data from patients normalized to average of controls (dotted line), as in **(A,B)**. **(E)** Representative immunoblots and protein gel. ^*^P < 0.05 by Mann-Whitney test.

Diaphragm mRNA levels of Nox2, p22^phox^, p47^phox^, p67^phox^, and p40^phox^ were increased with median values ranging from 2.5- to 4.6-fold over controls (Figure 1). The protein levels of Nox2, p47^phox^, p22^phox^, and p67^phox^ were also increased in diaphragm of HF patients, while protein levels of Rac1 was not significantly changed (Figure 1). We were not able to detect p40^phox^ via immunoblot in the diaphragm, which is consistent with a previous study (Javesghani et al., 2002).

**Figure 2:**
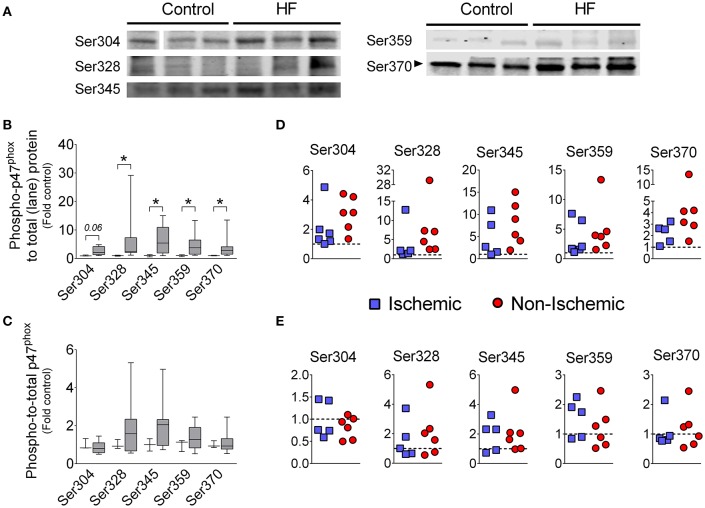
**Levels of serine phosphorylation of p47^**phox**^ in diaphragm of patients with end-stage HF. (A)** representative images of immunoblots, **(B,D)** levels of phosphorylated p47^phox^ normalized to total protein loaded into gel lane, **(C,E)** levels of phosphorylated p47^phox^ normalized to immunoblot signal of total p47^phox^ (from this figure). Controls (open boxes; *n* = 3) and HF patients (gray boxes; *n* = 9–11). Panels D and E are individual data from patients normalized to average of controls (dotted line)—y-axis labels as in **(B,C)**, respectively. Representative protein gels are similar to that shown in Figure 1. ^*^P < 0.05 by Mann-Whitney test.

Phosphorylation of p47^phox^ promotes activation of Nox2 (El-Benna et al., 2009; Lassègue et al., 2012), thus we examined the phosphorylation status of p47^phox^ using antibodies against specific phosphorylated serine residues. We found that phosphorylation at Ser328, Ser345, Ser359, and Ser370 were increased in diaphragm of HF patients compared to controls when normalized to total protein (Figure 2). When we normalized the phosphorylated signal from each serine residue to the total p47^phox^ signal, there was no difference in phospho-to-total p47^phox^ between control and HF patients (Figure 2). This suggests that the total abundance of p47^phox^ protein in the phosphorylated state was elevated, whereas the “percentage” of phosphorylated p47^phox^ was unchanged.

**Figure 3:**
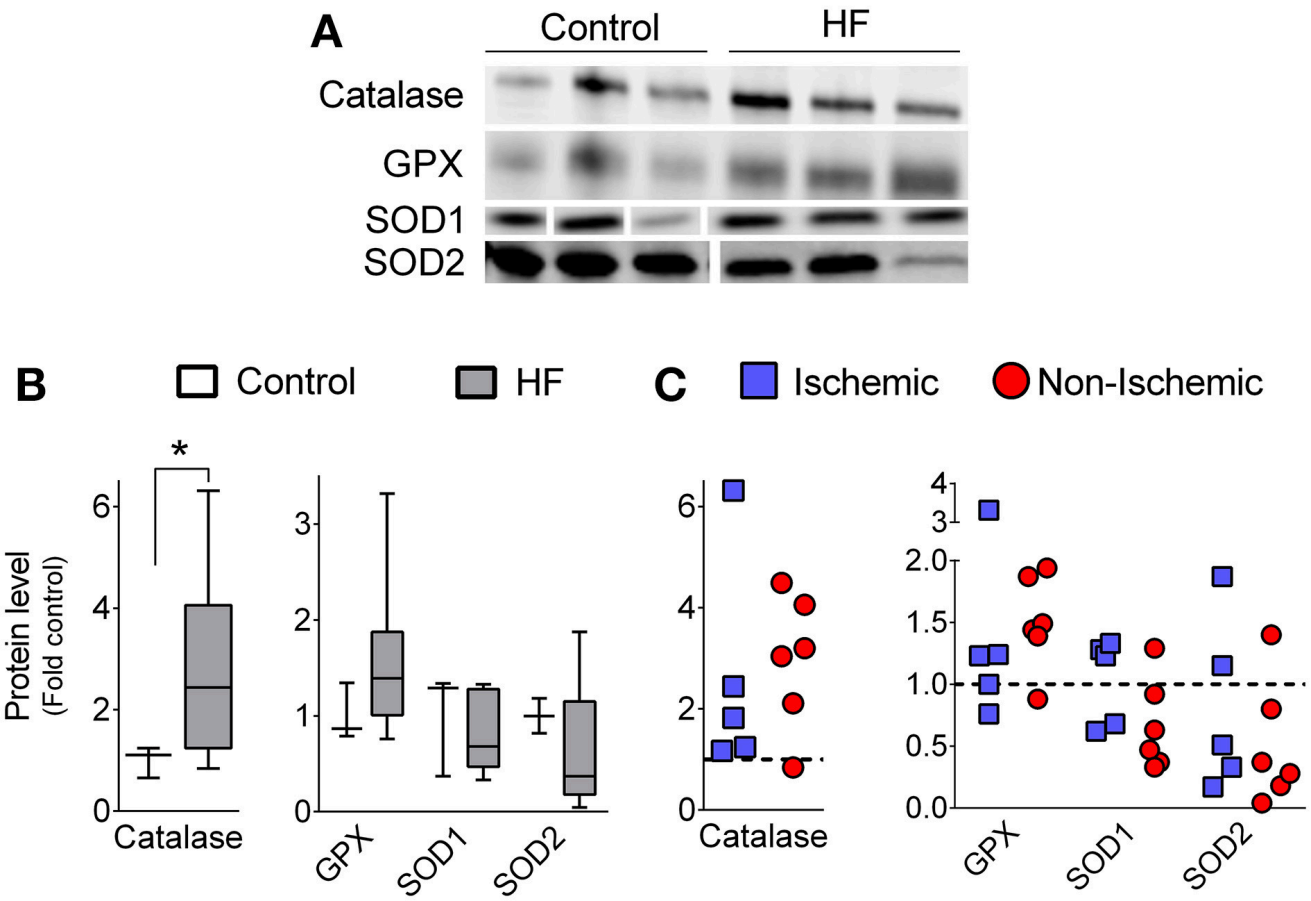
**Diaphragm protein levels of antioxidant enzymes**. **(A)** Representative immunoblots. **(B)** Grouped data from control (*n* = 3) and all patients with HF (*n* = 11). **(C)** Individual data from patients with ischemic and non-ischemic cardiomyopathy. Representative protein gels are similar to that shown in Figure 1. ^*^P < 0.05 vs. control by Mann-Whitney test.

We measured the protein level of key cytosolic and mitochondrial antioxidant enzymes (Figure 3). Catalase levels were increased 2.2-fold over controls (*P* < 0.05), whereas the levels of glutathione peroxidase (*P* = 0.15), SOD1 (*P* = 0.48), and SOD2 (*P* = 0.22) were unchanged in the diaphragm of HF patients.

**Figure 4:**
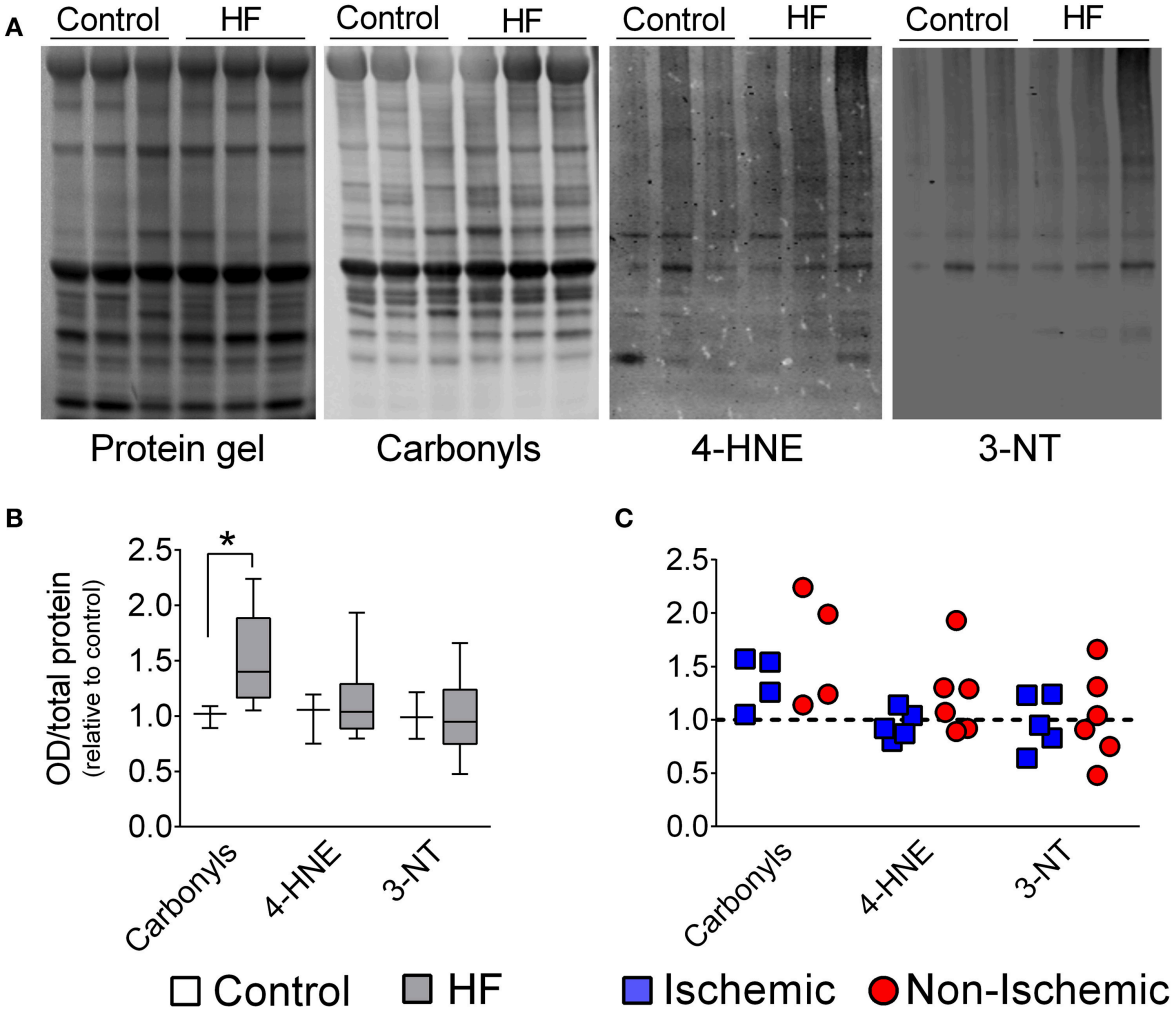
**Diaphragm protein oxidation in control and patients with end-stage heart failure. (A)** Representative lanes of protein gel, carbonyls, 4-hydroxynonenal (4-HNE), and 3-Nitrotyrosines (3-NT). **(B)** Box and whisker plot shows grouped data from control (*n* = 3) and all patients with HF (*n* = 8–11). **(C)** Scatter plots show individual data from patients with ischemic and non-ischemic cardiomyopathy. ^*^P < 0.05 by Mann-Whitney test.

Despite increased levels of catalase, redox imbalance in diaphragm from patients was manifested by increased (~40%) protein carbonyls, while 4-HNE and 3-NT were unchanged in the diaphragm of end- stage HF patients (Figure 4).

## Discussion

Our study shows that diaphragm of patients with end-stage HF have elevated mRNA and protein levels of Nox2 subunits that is accompanied by increased p47^phox^ phosphorylation, which is consistent with Nox2 activation. The antioxidant enzyme catalase was also increased in diaphragm of patients, while superoxide dismutases and glutathione peroxidase were unchanged. These findings suggest disrupted redox homeostasis in the diaphragm of patients with end-stage HF, which are confirmed by elevated levels of protein carbonyls.

The enzyme Nox2 is emerging as an important source of oxidants that cause diaphragm abnormalities in animal models of diseases, including muscular dystrophy (Whitehead et al., 2010; Pal et al., 2014; Henriquez-Olguin et al., 2015) and HF (Ahn et al., 2015). The functionally assembled Nox2 complex includes several subunits (Nox2, p47^phox^, p22^phox^, p40^phox^, p67^phox^, and Rac1 Bedard and Krause, 2007; Lassègue et al., 2012). We observed that mRNA levels of several Nox2 subunits were increased in the diaphragm of end-stage HF patients, with p47^phox^ subunit having the highest elevation (Figure 1).

Increased p47^phox^ mRNA was translated into higher protein abundance compared to control (Figure 1). Similarly, we have found heightened protein levels of p47^phox^ in the diaphragm of mice with HF (Ahn et al., 2015). It is unclear whether elevated p47^phox^ is sufficient to heighten Nox2 activity in skeletal muscle cells. Overexpression of p47^phox^ increases Nox2 activity in glial cells (Lavigne et al., 2001). Thus, it is possible that skeletal muscle cells have a constitutive, p47^phox^-dependent Nox2 activity.

The canonical pathway for Nox2 activation involves p47^phox^ phosphorylation at serine residues that releases auto-inhibition of membrane- and subunit-binding domains (El-Benna et al., 2009; Drummond et al., 2011; Lassègue et al., 2012). The C-terminal domain of p47^phox^ contains 11 serine residues between amino acids 303–379 that encompasses the auto-inhibitory region. Point mutations have revealed six serine residues of p47^phox^ that are required for full activation of Nox2: Ser303, Ser304, Ser328, Ser359, Ser370, and Ser379 (reviewed in El-Benna et al., 2009). We found that total levels of phosphorylated Ser328, Ser345, Ser359, and Ser370 were elevated in diaphragm of patients with end- stage HF (Figure 2). When we calculated the phospho-to-total p47^phox^ ratio, differences between HF and controls were not statistically significant (Figure 2C). These findings suggest that increased “absolute” levels of phosphorylated p47^phox^ accompanied the heightened expression of total p47^phox^. However, the “percentage” (or relative levels) of p47^phox^ protein in the phosphorylated state was unchanged. Typically, an increase in relative levels/percentage of p47^phox^ phosphorylation (i.e., elevated phospho-to-total p47^phox^) is considered an indicator of Nox2 activation (Isabelle et al., 2005). In the context of elevated levels of total p47^phox^, unchanged phospho-to-total p47^phox^ ratio should also heighten Nox2 activation because phosphorylated p47^phox^ proteins are more abundant. Biologically, the absolute amount of phosphorylated p47^phox^ would dictate Nox2 activity. Therefore, we consider that Nox2 activity is likely increased in diaphragm of end-stage HF patients. However, the unchanged relative levels/percentage of phospho-p47^phox^ has implications regarding mechanisms of Nox2 activation. Our data suggest that the activity of kinases that phosphorylate p47^phox^ is not necessarily elevated in diaphragm of patients with end-stage HF. It is possible that other pathways of p47^phox^ signaling (e.g., arachidonic acid) or overexpression *per se* mediate Nox2 activation in the diaphragm (Ferreira and Laitano, 2016). Alternatively, enhanced diaphragm p47^phox^ phosphorylation may be involved in the pathophysiology of diaphragm dysfunction at earlier stages of the disease.

We have not tested Nox2 activity in our study because our tissue collection method (flash freezing) does not lend the sample suitable for reliable measurements of activity, as per recent recommendations (Rezende et al., 2016). We have found, in diaphragm of mice with HF, increases in total p47^phox^ and phospho-to-total p47^phox^ that are consistent with increases in Nox2 activity (Ahn et al., 2015). Indeed, knockout of p47^phox^ prevented excess diaphragm ROS emission suggesting Nox2 as a major source of pathological diaphragm oxidants in HF (Ahn et al., 2015). Overall, our data in humans and animals suggest elevated Nox2 activity in patients with HF.

A decrease in protein levels or intrinsic activity of antioxidant enzymes will contribute to ROS accumulation that disrupts cellular redox balance. Major intracellular antioxidant enzymes include catalase, glutathione peroxidase, and superoxide dismutases (SOD1 and SOD2). Patients had increased levels of catalase, whereas there was no statistical difference in the levels of GPX, SOD1, and SOD2 between patients and controls (Figure 3). This outcome is likely due to our limited sample size and large variability in the human diaphragm data. In animal models of HF with reduced ejection fraction, diaphragm levels of SOD1 or SOD2 were either unchanged (Ahn et al., 2015; Laitano et al., 2016) or elevated (Mangner et al., 2015), whereas the activity of GPX was increased (Mangner et al., 2015) and catalase was unchanged (Mangner et al., 2015). Nonetheless, diaphragm catalase activity was increased in a model of HF with preserved ejection fraction (Bowen et al., 2015b). In general, our data in patients and studies in animals suggest that heightened protein oxidation in the diaphragm induced by HF cannot be explained by a decrease in the protein levels or activity of antioxidant enzymes. Heightened diaphragm antioxidant enzyme levels in HF might be a compensatory adaptation aimed to maintain redox balance when ROS production is increased. However, our findings suggest that any compensatory response is insufficient to maintain normal protein oxidation levels in patients with end-stage HF.

Protein carbonyls, a marker of oxidation, were elevated in the diaphragm of end-stage HF patients (Figure 4). These results corroborate previous findings in animals with severe HF induced by aortic stenosis (Coirault et al., 2007) or in the early stages post-myocardial infarction (Supinski and Callahan, 2005; Bowen et al., 2015a), but disagrees with data from our group in rats and mice with moderate HF in the later stages post-myocardial infarction (Ahn et al., 2015; Laitano et al., 2016). We speculate that the increase in diaphragm protein carbonyls in chronic HF is related to disease severity. This concept is supported by progressive increases in systemic markers of oxidation in groups of patients going from NYHA Class I to IV (Belch et al., 1991; Nishiyama et al., 1998).

Excess oxidation can contribute to diaphragm abnormalities due to protein degradation as well as impaired function of excitation-contraction coupling and sarcomeric proteins. Oxidation enhances protein degradation by calpain, caspase-3, and the proteasome (Grune et al., 2003; Moylan and Reid, 2007; Smuder et al., 2010). Calpain and proteasome activity are elevated in diaphragm of HF rats (Dominguez and Howell, 2003; van Hees et al., 2008a), and proteasome inhibition prevents myofibrillar protein degradation and attenuates diaphragm weakness in HF rats (van Hees et al., 2008a). Regarding protein function, carbonylation of sarcomeric proteins *in vitro* impairs actomyosin cross- bridge kinetics and, in general, exposure to oxidants depresses diaphragm force and shortening velocity (Perkins et al., 1997; Callahan et al., 2001; Coirault et al., 2007). These functional outcomes are consistent with impaired diaphragm contractile properties in HF animals (Lecarpentier et al., 1998; Coirault et al., 2007; van Hees et al., 2007, 2008b; Empinado et al., 2014; Ahn et al., 2015), which are prevented by pharmacologic antioxidants (Supinski and Callahan, 2005; Laitano et al., 2016) or knockout of p47^phox^ (Ahn et al., 2015).

### Limitations

There are several limitations in our study that must be considered for data interpretation. These limitations include: (*A*) *age and sex of controls and patients:* Patients in the HF group were mostly males with both ischemic and non-ischemic cardiomyopathy, while controls were all females and younger than HF patients. Inspiratory muscle weakness and diaphragm abnormalities are relevant for patients with ischemic and non-ischemic HF (Ambrosino et al., 1994; Lindsay et al., 1996; Tikunov et al., 1997; Daganou et al., 1999; Filusch et al., 2011) as well as male and female patients (Ambrosino et al., 1994; Lindsay et al., 1996; Dall'Ago et al., 2006). We did not have sufficient number of patients with ischemic and non-ischemic cardiomyopathy to resolve potential statistical differences due to etiology of disease. Inspection of data from patients in each group suggest that overall the changes were consistent for both ischemic and non-ischemic cardiomyopathy. Nonetheless, in this data set there is a general trend for exacerbated effects in patients with non-ischemic cardiomyopathy. The data from the female HF patient were consistent with those from males. For instance, p47^phox^ levels from the female HF patient corresponded to 9.5-fold (mRNA) and 4.5-fold (protein) of the control mean. The lack of age- and sex-matched data in humans reflects the nature of our study and focus on the diaphragm that presents difficulty for obtaining biopsies, especially from control subjects. (*B*) *Mechanical ventilation:* Brain-dead organ donors undergo mechanical ventilation, which heightens diaphragm protein oxidation (Betters et al., 2004). We do not have information on the duration of mechanical ventilation in our control subjects. It is possible that controls underwent longer periods of mechanical ventilation than our HF patients experienced during surgery. However, this would minimize rather than accentuate differences in the variables that we studied. (*C*) *Inability to establish cause-and-effect:* we cannot establish a causal relationship between Nox2 levels/activity, protein oxidation, and diaphragm abnormalities in HF patients. Instead, our data should serve as an impetus for clinical trials testing selective Nox2 inhibitors or pharmacological antioxidants to treat diaphragm abnormalities and its associated complications in end-stage HF patients.

It is worth noting that important studies relying on diaphragm biopsies of end-stage HF patients and controls also had an unbalanced distribution of age or sex and included brain-dead controls or patients with HF due to several causes (Lindsay et al., 1996; Tikunov et al., 1996, 1997). Finally, we cannot attribute our findings to proteins within diaphragm muscle cells *per se*. In addition to muscle fibers, several other cell types within the diaphragm express Nox2 subunits, e.g., endothelium, smooth muscle, and macrophages.

## Conclusion

Diaphragm of patients with end-stage HF shows upregulation of Nox2 subunits, increased total but unchanged relative levels of phosphorylated p47^phox^, and elevated abundance of catalase. These changes in ROS-producing and scavenging enzymes culminated in elevated diaphragm protein oxidation. Overall, our findings suggest that Nox2 is an important source of ROS in the diaphragm of patients with end-stage HF and increases in catalase levels are not sufficient to maintain cellular redox homeostasis.

## Author contributions

Sample collection and processing: JM, CH, MB, and LF. Experiments: BA, PC, AB, JM, NP, and LF. Data analysis and interpretation: BA, PC, AB, JM, NP, MB, CH, AJ, and LF. Manuscript writing and editing: BA, PC, AJ, and LF. Patients assessment and surgeries: CH and MB.

## Funding

This study was funded by NIH grant R00-HL098453 to LF. AJ was funded by NIH grant R01 AR060209.

### Conflict of interest statement

The authors declare that the research was conducted in the absence of any commercial or financial relationships that could be construed as a potential conflict of interest.
